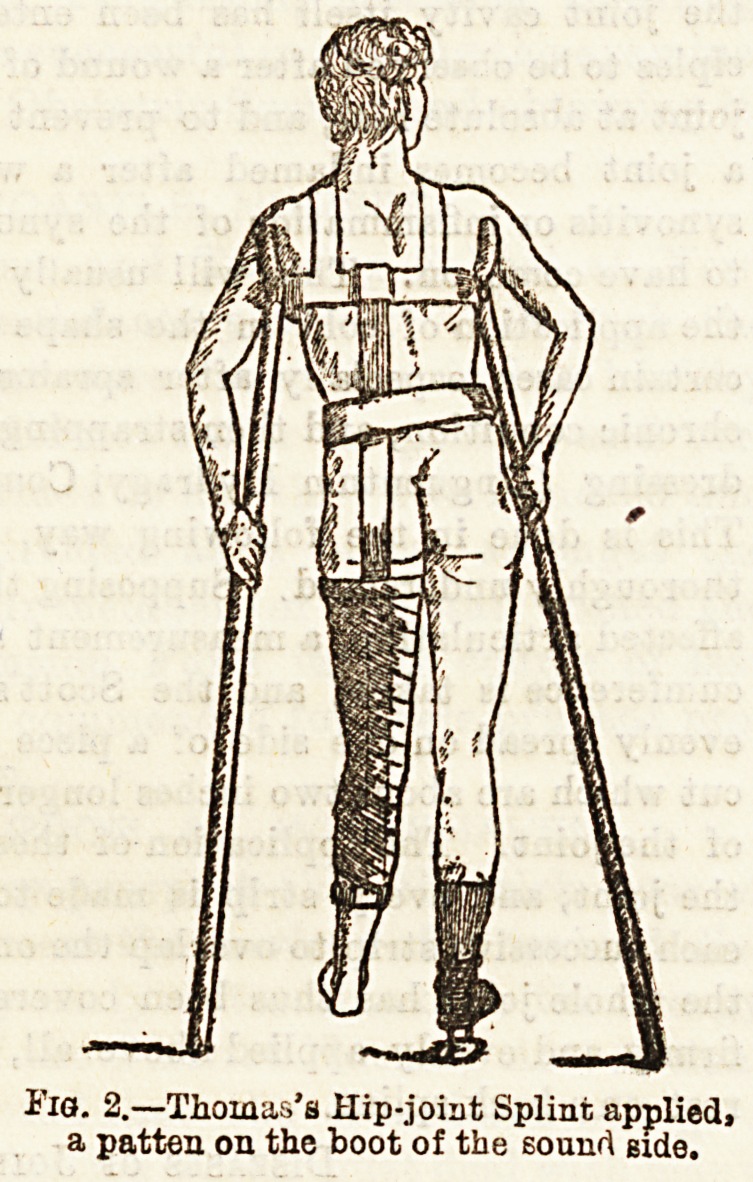# The Hospital Nursing Supplement

**Published:** 1895-04-06

**Authors:** 


"The Hospital" Nursing Mirror.

Being the Extra Nursing Supplement of "The Hospital" Newspaper.

[Contributions for this Supplement should be addressed to the Editor, The Hospital, 428, Strand, London, W.C., and should have the word "Nursing" plainly written in left-hand top corner of the envelope.]

### News from the Nursing World.

### ROYALTY IN NORTH LONDON.

The Great Northern Central Hospital was honoured by a visit from H.R.H. the Duchess of York last week. The senior physician, Dr. Robert Burnet, and Mr. J. Macready, the senior surgeon, accompanied the Duchess round the wards. She was attended by Lady Eva Greville and Sir Francis de Winton. The announced improvement in the health of H.R.H. the Duke of York has been gladly noted, and we hope soon to hear of his complete convalescence.

### AT PADDINGTON GREEN.

The bazaar opened on March 28th by the Countess of Warwick, at the Paddington Green Children's Hospital, was an unqualified success, being thronged with visitors during three days. The entertainments were numerous, and included plays, concerts, and skirt dancing. Madame Gomez, Miss Mary Moore, and Madame Antoinette Sterling were among the vocalists. The new building, although unfinished, gives every promise of being one of the prettiest children's hospitals in London, and the opening of the extensive wards is anticipated with much interest. The walls are adorned by pictures formed in tiles, the designs being most artistic, and the subjects such as will be fully appreciated by the children. Much good work has been done in the old building, and there is no fear that the new one will be any less popular, whilst modern fittings and conveniences will greatly facilitate administration. The nurses present at the bazaar were remarkable for the neatness of their uniform, and their pretty caps were specially noteworthy.

### BAD TIMES FOR BABIES.

Deplorable is the report made by Dr. Valleance on the condition of the West Ham Schools. He informs the Guardians that fifty-three sick children are under the care of one day nurse, and have no night nurse at all. Overcrowding has raised the number of inmates from the 260 sanctioned by the Local Government Board up to 400, and according to the local press report, the bedsteads now touch one another. Attention was drawn to the entire absence of means for isolating infectious cases, and also to the immediate presence of whooping cough. The Guardians will do well to remedy these evils ere some serious epidemic sets in.

### THE S.P.C.K. MEDICAL MISSION.

On March 14th a well-attended meeting of the Medical branch of the S.P.C.K. was held, and a report given of the work of the branches in India, Palestine, China, Japan, and North America. The Bishop of Caledonia gave eloquent testimony to the help received from the society, a doctor and two trained nurses being maintained at the settlement of Metlakatla, in his diocese, where English and Indians have equal attention. An address was also given by Dr. Alice Marston, of the North China Mission, and Mr. Fors described the Training Institute for Nurses at Kobe, which receives native women. The natural gift for nursing possessed by the Japanese women appears to make them most satisfactory pupils.

### LEWISHAM INFIRMARY.

Last week we announced that Miss Patteson had returned to her duties at the Lewisham Infirmary on the 20th ult. In the report of the proceedings at the meeting of the Lewisham Guardians on March 18th which appeared in The Hospital of the 23rd ult., on page clxxxiii., it was intimated that something more would transpire at the next meeting. This was held on the 1st instant, and as the question of the matron was not alluded to, we hope that matters will now be allowed to take their course, and that everything will work smoothly and happily at the Lewisham Infirmary in future.

### A LITTLE DISPENSING.

"She must understand a little dispensing," remarked some one, enumerating the qualifications needful for a cottage hospital matron. Apparently it did not occur to the speaker that there was any incongruity in demanding from candidates for the vacant post full training in nursing, and thorough knowledge of housekeeping, whilst a slight acquaintance might suffice in the other branch of her duty. However, it is probable that the responsibilities of cottage matrons regarding drugs would be largely shared by an experienced local doctor, and being a fully-trained nurse, there should be no fear of her venturing any prescriptions on her own account. In certain religious communities, however, great courage seems allied to small knowledge, the worthy sisters prescribing as well as dispensing free physic to their poorer adherents, although possessed of no qualifications for practising either medicine or dispensing.

### SOCIETY OF TRAINED MASSEUSES.

The first examination held by this society appears to have been conducted in a very thorough manner, the papers of the candidates being submitted to an independent authority, as also was the practical and *viva voce* examination. Seven candidates passed successfully and were placed on the roll of the society, and, after having subscribed to its rules, they received certificates. The address of the Hon. Secretary is 12, Buckingham Street, Strand.

### BETTER LATE THAN NEVER.

The grievances regarding the nursing, or rather the lack of it, at Bath Workhouse Infirmary are likely, at last, to receive due consideration. The Hospital Committee have recommended the Guardians to follow the advice of their medical officer (which coincides with that of the Local Government Board), and to engage nine nurses. They also recommend that the superintendent nurse be first secured that she may assist in organising the staff under her. Our readers will remember that it was at first proposed by the committee that the wishes of the Local Government Board should be carried out only in a modified form, but they have now wisely decided to recommend the adoption of the proposed reforms in their entirety, and the sick inmates will certainly benefit by this humane decision.

### PROBATIONERS AT BRISTOL.

At the Bristol Royal Infirmary, maternity work has been included in the training of probationers during the last two years, greatly to their own advantage, as well as for the benefit of the patients in the out-door lying-in department. No less than 4,000 visits were paid in the course of the year to 425 maternity cases, and Nurses Bygott, Fergusson, Matthias, Spurling, and Townsend were successful in passing the examination of the London Obstetric Society and obtaining its diploma.

### NEWCASTLE NURSES.

The Nurses' Home and Training School at Newcastle has issued a satisfactory report. Although the cases nursed last year were fewer than in less healthy seasons, the nurses did not suffer in pocket, and certainly must have gained in health. Two hundred and fifty pounds was divided as a bonus amongst the nurses. Twenty-four members of the staff joined the Royal National Pension Fund in the course of last year, and are to be commended for their prudent provision for the future.

### THE SICK POOR AT WHITEHAVEN.

There seems every prospect that the sick poor of Whitehaven will possess the services of a trained district nurse at a very early date, thanks to the energy of the committee of the District Nursing Association. It is naturally expected that the Guardians will give support to an organisation which must deal with many cases which would otherwise come upon the rates, and several members of the Board appear to realise their obligations. At a recent meeting, attention was drawn to the value of a trained nurse's services in the homes of the destitute sick, and to the obvious economy of assisting the bread-winners to regain their health with as little delay as possible. However, the Whitehaven Guardians, as a body, appear fearful of committing themselves, before seeing exactly what they will gain by a subscription to the new association, and they, moreover, profess ignorance as to the Local Government Board sanctioning such expenditure. The Whitehaven Guardians seem unaware that in many places subscriptions have already been voted for similar objects, and that they are also empowered by a special order of the Local Government Board to engage and remunerate trained nurses as district nurses for the out-door poor. That but few Unions avail themselves of this privilege seems to prove that co-operation with existing district nursing societies is generally found satisfactory, subscriptions being readily sanctioned by the Local Government Board.

### NOTTINGHAM.

At the annual meeting of the Nottingham Samaritan Hospital the excellent work of Miss Paget and her nursing staff was cordially acknowledged. The meeting, which was presided over by the Mayor, was well attended, and suggestions as to the feasibility of amalgamation with the other Women's Hospital received due consideration.

### INADEQUATE REMUNERATION.

A scheme for the nursing of the North Infirmary, Cork, has appeared in the local press, and a report, prepared by a sub-committee, has been laid before the trustees of the institution. A home for the nurses is to be erected over the hospital laundry (not quite the most desirable of sites), and probationers are to be admitted on payment of £5 for the first two months, and a further sum of £25 if they remain on. After twelve months as probationers they are bound to remain in the service of the institution for three additional years, either in the hospital or at outside work, and during that time are paid £12 for first and £15 for second and £18 for third year. A lady superintendent will receive £40, and a night superintendent £20 per annum. It is, however, hardly likely that an efficient nurse will be found to take the latter post at so low a salary.

### DISTRICT NURSING IN WALES.

The Mayor of Denbigh took the chair at the annual meeting of the District Nursing Association on March 15th, and a satisfactory report was presented to the subscribers. The association is affiliated with the Queen's Jubilee Institute, and the nurse has had 180 patients under her care during the last year, to whom she has paid 2,896 visits. Miss Oldham, inspector of the Rural Branch of the Institute, reports most favourably on the work done at Denbigh. A vote of thanks was moved at the meeting to Nurse Jones, whose efficiency is cordially appreciated by doctors, patients, and committee.

### SHORT ITEMS.

Ruthin is to have a Queen's Nurse for the district. —In opening the recent sale of work in aid of the Hanley Nursing Society, the Duchess of Sutherland expressed her deep sympathy in everything to do with nursing.—At a meeting of the Colchester Guardians the absence of a proper night nurse at the infirmary was mentioned, also the deplorable fact that two old women, who (according to the local press report) had to work during the day, took it in turns at night to look after a patient who was almost at the point of death.—The probationers at Guy's Hospital now have two weeks holiday during their first year, three weeks in the second, and four in the third and following years. Twenty-five members of the nursing staff successfully passed the examinations held in February in materia medica, pharmacy, and dispensing.—A woman doctor has been appointed divisional surgeon to the Northern Pacific Railway at Hope Idaho; her name is Miss Carrie Liebig.—The *St. Thomas's Hospital Gazette* for March contains a very able article on "Celebrated Midwives of the 17th and thebeginning of 18th Century, and on the Present Position of Midwives."—The Devonport Guardians have decided against the appointment of the proposed ladies' visiting committee. —The funds of the Cottage Nursing Association at Wotton-under-Edge have benefited by the proceeds of two excellent concerts, given at Alderley House by the kind permission of General Hale.—The Penzance Guardians have now decided that instead of complying with the recommendation of the Local Government Board respecting a certificated nurse for the sick, they will merely advertise for "a young woman."—The last of the winter concerts at Paddington Workhouse was given on March 26th by Miss Tryphena Hanney.—It is reported by the press that a Local Government Board inquiry has been demanded relative to the recent "suprise visits" to St. Olave's Infirmary; one of the nurses has addressed a letter to the Guardians protesting against certain remarks made at their last meeting.—The Frome Home for Trained Nurses has issued its eleventh annual report, it provides private nurses for the well-to-do, and district nurses for the poor.

### Elementary Anatomy and Surgery for Nurses.

By W. McAdam Eccles, M.B., M.S., F.R.C.S., Lecturer to Nurses, West London Hospital, &c.

### XI. INJURIES OF JOINTS.—DISEASES OF JOINTS.

There are three chief injuries to joints to which attention must be called. They are: (1) Dislocations, (2) Sprains, (3) Wounds.

(1) Dislocations.—A joint is said to be dislocated when the surfaces of the bones which are normally in contact with each other, thus constituting an articulation, are separated from one another. Dislocations are the outcome of violence, disease, or congenital malformation. Most of the joints of the limbs may be dislocated, and we have seen in a previous lecture that fracture of the vertebral column is most often associated with dislocation.

*Dislocation of the Shoulder Joint.*—When the mobility of this joint, and the disproportion between the head of the humerus and the glenoid cavity of the scapula, is considered, it will be readily expected that it is very prone to dislocation. When they have been reduced by the surgeon the bones will need to be retained in place by bandaging the arm to the chest. Passive movement will be needed after the first ten days.

*Dislocation of the Elbow Joint.*—Most commonly both bones —radius and ulna—are dislocated backwards. When reduced an internal angular splint is to be applied. There is usually very great effusion into the joint. A good deal of stiffness may result, so that gentle movements are to be commenced early. Dislocations of the other joints of the upper extremity are not very common.

*Dislocation of the Hip Joint.*—From the configuration of this almost perfect ball and socket joint it will be surmised that dislocation is but rarely sustained, except from severe violence. After reduction it is usual to keep the joint at rest with a Liston's long splint for about a fortnight. Dislocation of the knee joint is uncommon, and requires no description here; but

*Dislocation of the Ankle Joint* is frequently met with combined with fracture of the fibula within three inches of the external malleolus. The whole foot is displaced at the ankle joint outwards, backwards, and upwards, at the same time the tip of the internal malleolus is torn off, or the internal lateral ligament of the ankle joint ruptured. The entire lesion constitutes a Pott's fracture. This may be treated in the same manner as was mentioned for fractures of the leg, *i.e.*, by a box splint and a Salter's cradle. Sometimes, however, an outside splint with a foot piece will be more advantageous.

*Dislocation of the Lower Jaw* is a fairly common displacement caused by yawning, laughing, &c. The bone may slip out either on both sides or only one. It is usually comparatively easily reduced, and then kept in place by a four-tailed bandage, the patient for a fortnight taking only liquid and soft food.

(2) Sprains.—When violence is applied to a joint, and no dislocation results, there is often much stretching and even laceration of the ligaments, with the consequence that great swelling of the part occurs, with exquisite tenderness and pain on movement. Some sprains are undoubtedly worse in their results than even dislocations. If a sprain be sustained and the joint seen before much swelling has come on, firm bandaging with a water-wetted bandage will be the best treatment, though in the more severe cases plaster of Paris bandages applied over a thick layer of cotton wool will be more efficacious. If the effusion has become well marked, and the pain is great, hot or cold applications will give great relief. In the stiffness which is so prone to follow after a sprain, shampooing, rubbing, and passive movements will be found to be of the greatest benefit. More forcible movements under an anæsthetic may be required in some cases.

(3) Wounds. — These naturally differ very much in character, and in the extent of damage which the joint proper sustains. Punctured wounds are certainly the most dangerous, seeing that they are liable to be followed by septic inflammation of the joint. It is a good rule to follow, that any punctured wound near a joint should be treated as a wound of the joint, although there may be no direct evidence that the joint cavity itself has been entered. Two great principles to be observed after a wound of a joint are, to keep the joint at absolute rest, and to prevent septic infection. When a joint becomes inflamed after a wound or other injury, synovitis or inflammation of the synovial membrane is said to have come on. This will usually subside with rest and the application of cold in the shape of an ice-bag. But in certain cases, especially after sprains, it is apt to become a chronic condition, and then strapping the joint with Scott's dressing (Unguentum Hydragyi Compositum) is advisable. This is done in the following way, which a nurse should thoroughly understand. Supposing the knee-joint to be the affected articulation, a measurement around its greatest circumference is taken, and the Scott's dressing having been evenly spread on one side of a piece of moleskin, strips are cut which are about two inches longer than the circumference of the joint. The application of these is commenced below the joint, and every strip is made to overlap in front, and each successive strip to overlap the one next below it. When the whole joint has thus been covered a bandage should be firmly and evenly applied above all, and the joint kept at rest on a back splint.

### Diseases of Joints.

The most important disease of joints, and indeed the most frequent is one which is produced by the parasite known as the tubercle bacillus. This is a very minute rod shaped micro-organism belonging to the vegetable kingdom and causing great havoc when it settles in the tissues of the human body. It is the essential cause of consumption. Such joint disease is far more frequent in children than in adults. Almost any joint in the body may be thus affected, though there is a marked tendency for the parasite to settle in the hip, knee, or elbow. Normal healthy living tissues are able to resist the invasion of the bacillus, but if the part is below par, or has been the seat of some injury, often a very trivial one, it is liable to become infected. At first there is some obscure pain, especially on a movement of the joint. Then a characteristic alteration in the shape and position of the parts occurs. Now it is, that if complete rest be afforded the joint, there exists a great probability of entire restoration to health, but at the same time there is a grave danger of abscesses forming, and of the joint being absolutely destroyed with considerable deformity and loss of function resulting.

No cases, perhaps, need greater care and patient attention on the part of nurse and surgeon alike than those of hip joint trouble in children, and none perhaps require it for a more extended period of time but in the end so well repay the labour spent. A child requires all the resources a nurse can bring to bear when it is ill and suffering. It does not cry from pure wickedness, which a healthy child may. Its little body is weary of the continuous lying on its back, it is unable to express its wants, and its aches and pains are real though perhaps unnoticed by its elders. Win the confidence of your child patient, and half the battle of the successful nursing of the little sufferer will be won. Be the child's confidant, companion, friend, as well as nurse. The general treatment of an early case of hip disease is to keep the affected limb at rest in an extended position, with an extension apparatus such as was mentioned in the last lecture. If abscesses have occurred and have been opened, it is of the utmost importance to see that the dressings do not become fouled in any way. A Thomas's hip splint is one which is frequently used, for while it keeps the joint at rest it allows the patient to be out of bed, and thus able to get into the open air.

By reference to Figs. 1 and 2, it will be seen that this splint consists of an upright iron piece which reaches from about the level of the lower angle of the scapula, to just below the prominence formed by the calf muscles, and is moulded to fit the curves of the trunk and limb it meets with. Three circular pieces of flexible iron will also be observed, one at the upper-

Fig. 1.—Thomas's Hip-joint Splint.

Fig. 2.—Thomas's Hip-joint Splint applied, a patten on the boot of the sound side.

most part of the splint to partly encircle the chest, one to surround the thigh, and the third the leg. The nurse will have to see that this splint is thoroughly well padded and covered with chamois leather, and in addition fitted with buckles and straps. In order that no pressure on or jarring of the diseased hip may occur the patient has a patten applied to the boot of the sound limb, and walks with the aid of crutches.

In disease of the spine a double Thomas's splint is often a most useful apparatus. Space does not allow of any fuller reference to these deeply interesting cases of tubercular disease of joints.

### Nursing in France.

Not only in Paris is sick nursing taught in classes. Bordeaux also rejoices in its free school. Theory must in such circumstances largely take the place of the practical experience which has long been acknowledged as the only adequate preparation for qualified nurses.

Bordeaux Free School for Nurses.

In 1884 the Protestant Lunatic Asylum at Bordeaux inaugurated a series of free public lectures, to dispense knowledge, combat popular prejudices, and minimise harm to the sick or injured pending the arrival of the doctor.

The lectures are given in a practical, popular manner by a member of the Medico-surgical Committee.

In 1887 those who had attended the course regularly, were admitted to the clinical hospital of the asylum. In November, 1890, the administrative council, at the suggestion of the doctors of the asylum, voted for the foundation of a free school for nurses, and from that date the doctors have given constant lectures at the school. The free public lectures have also been continued regularly every year.

In order to become a pupil at the school, candidates must be at least eighteen years of age, be perfectly respectable, and have a sufficiently good elementary education. Admissions are irrespective of creed, the aim of the school being humanitarian and unsectarian.

The course of instruction lasts two years. At the end of the first year, if the pupils have satisfactorily passed the four preliminary examinations, they are admitted to the second year's training, at the end of which those who have passed the final examination receive their diplomas. In July the directress commences practical demonstrations to the first year pupils in turn, and on different days to the second year pupils.

Those desirous of profiting by the lessons without being either questioned or examined are permitted to be present.

The nursing diploma was obtained on February 18th, 1893, by eight pupils, and by eleven pupils in the July following. In July, 1894, eleven more pupils received the diploma, after a final examination.

The candidates were questioned on anatomy, physiology, antiseptic treatment, hygiene, contagious diseases, elementary surgery, surgical pathology, symptoms which a nurse must note in order to report to the doctor, bandagings, dressings, and the naming of instruments.

It is evident that this scheme aims at bringing knowledge, in a popular form, within reach of all. The title of nurse, however, should not be conferred on any pupil who has only attended classes and demonstrations, and had no hospital training whatever.

### Presentations.

The committee and medical staff of the Cottage Hospital, Bromley, Kent, presented Miss Heather-Bigg, on her resignation of the post of matron, with a handsome clock and lamp. A silver fruit knife, butter knife, and watch case were given by the nurses and servants, with many expressions of good-will.

### Notes and Queries.

### Queries.

(111) *The Daily Nurse.*—Can you tell me whether a plan, discussed some time since in The Hospital, of nurses visiting several patients daily, and staying an hour or more with each, has ever been carried out?—*I. H. M.*

(112) *Training.*—I should be glad to know of a good hospital or infirmary where a lady could be trained gratis? She is 35, and in every respect suitable.—*E. C.*

(113) *Private Nursing.*—Can you tell me anything about private nursing in Edinburgh?—*Querist.*

(114) *Australia.*—Can you tell me who could inform me about district nursing in Sydney or Melbourne?—*Nurse H. M. J.*

### Answers.

(111) *The Daily Nurse (I. H. M.).*—We have met with nurses who have carried out this idea, but they find a little difficulty in persuading patients who engage their services for one hour a day that they must be attended on in rotation. Certain times of the day being obviously more convenient to the household than others, the nurse has to use her discretion in arranging her hours to suit doctor and patient, and also to fit in with her other engagements. Many nursing institutions charge a regular fee for the single visits of their nurses, for attendance at operations, &c. From your letter we imagine it is a kind of fully-trained district nurse for paying patients which you require. Miss C. J. Wood, Nurses' Hostel, 27, Percy Street Tottenham Court Road, would probably be able to help you in this. You would also obtain full particulars as to the cost from the lady superintendent of the Nurses' Co-operation, 8, New Cavendish Street, London.

(112) *Training (E. C.).*—We do not think a lady of 35 would be received as a general probationer in a London institution. Possibly in a fever or provincial hospital she might be accepted. She should write to a number of matrons and ask for rules. In "Burdett's Hospital Annual" she will find a list of training schools.

(113) *Private Nursing (Querist).*—There is no branch of the Nurses Co-operation in Edinburgh. Therefore, you would have to rely for getting work entirely on the doctors, to whom, of course, you would need personal introductions. Do not lose sight of the fact that most excellent nurses are trained in Scotland, so you would have to compete against many equally competent women.

(114) *Australia (Nurse H. M. J.).*—Miss McGahey, Matron of the Prince Alfred Hospital, Sydney, could give you the information you desire. She has done much for nursing out there. You are, probably, aware that there is comparatively little demand for English nurses, now that so many colonies train their own. You should not attempt to go out unless you have independent means, and are also prepared to encounter considerable opposition. The Colonial trained nurses do not welcome English rivals.

### Asylum News and Nursing.

[Contributions to this section should be addressed to The Editor, 428, Strand, W.C., and have the words "Asylum News" written in left-hand bottom corner of the envelope.]

### LETTERS FROM AN ASYLUM NURSE.

### A Day's Work.

In this letter I intend giving an account of the daily life and duties of an under nurse in an asylum. The hours on duty, usually from six a.m. in summer or half-past six a.m. in winter to eight p.m., are long, and it is no wonder some women find the occupation very trying. The wages for the various classes of nurses (I am not speaking of head nurses) run from about £18 to £33 yearly, with uniform, &c. At some asylums a bonus at the end of a certain period is given, as, for example, £5 at the end of every five years, and there is always a fair prospect of a pension after long service. The nurse on day duty is, of course, free in the evening, as a rule, from eight to ten. She has about two and a half days' leave in a month, including one Sunday, and the annual holiday varies from ten to fourteen days. I will give a sketch of my day's work at a time when, after a few months, I was given charge of a dormitory, and will suppose it was during the summer, the hours in winter being rather different. At six o'clock I went to the dormitory with the charge nurse to see that the patients were all right before signing the night-book, and thus becoming responsible for them. The patients' clothes were then given to them, and those unable to look after themselves were dressed. The women capable of attending to their own toilet did so in the dormitory; those who required help were taken to the adjoining lavatory. The dormitory windows were opened, the beds turned down, and any slop on the floor dried up. This occupied us until about seven o'clock, and the patients were taken to the day-room, being counted on the way, and reported to the charge nurse as all right. She was by no means backward in expressing her opinion if she considered that any of them had not received proper attention. I then went to breakfast, and on my return relieved in the ward during second mess. The patients had breakfast at eight, and I had charge of a table in the dining-room. It was my duty to help the patients who sat at it, to take notice how they took their food, and to report anything unusual to the charge nurse. As a rule I had also a special case who required feeding and more particular care.

After breakfast I took a party of patients to the dormitory, some of these being recoverable cases, whom I had to look sharply after and to try and persuade to employ themselves. Before the slops were emptied I saw all the chambers, and if their contents were in any way unusual they were kept, and the matter reported to the charge nurse. A note was always made of any bed that was found wet, and the night nurse generally heard of it. My dormitory was not usually finished until eleven o'clock, when my patients were handed over to the nurses on the ward, and I had half an hour to myself in which to dress and snatch a little lunch. On my return I found the other nurses had got the patients ready for exercise, and I went with them often with a special case on my arm. About a quarter-past twelve we came in, and prepared for patients' dinner, at which my duties were much the same as at breakfast, with the addition of having to count the knives and forks before handing them to the nurses in charge of the dining-room. The nurses dined after the patients. In the afternoon, when fine, the patients were again taken out; when it was wet they were employed or amused indoors. At seven p.m., after patients' tea, I went to the dormitory to see to windows, shutters, &c., and except on evenings on which there were entertainments, at half past seven we began to put the patients to bed. Those unable to attend to themselves had to be undressed, and their clothes folded in little bundles ready for the morning. Wet and dirty cases required to be specially looked to. At eight p.m. the night nurse came on duty, and, unless under exceptional circumstances, I was free to go out.

### HOLIDAYS.

If there be any class of workers to whom a holiday is an absolute necessity, it is they who spend their lives in direct contact with the insane, and who are daily harassed with all the cares and anxieties which such an occupation entails. Every charge nurse and attendant is only too familiar with the state of mind in which they feel that they can no longer bear up under the strain, and at times the horrible thought will come that the work is becoming too much for them. The responsibility of looking after suicidal and dangerous cases is a heavy one, and the outside public is little disposed to give any credit for the efforts made. So long as everything goes right, those in charge are forgotten, but once let an accident occur, and we see how little sympathy is given to men and women who have devoted themselves to the hardest and most thankless branch of the noble work of nursing. No doubt many weary toilers are congratulating themselves that the holiday season is approaching, and that they will once again enjoy a well-earned respite from their anxious duties. We would urge upon them the necessity of obtaining a complete change from their usual surroundings. In these days of co-operation and self-help, why should not some energetic members of our asylum staffs organise trips for their fellow-workers? They could be done cheaply, and the holiday is quite long enough to allow even a short Continental excursion.

### INADEQUATE NURSING AND THE WORKHOUSES.

Attention has been called by the city coroner to the lunatic department of the Belfast Workhouse, which contains some 250 persons, and has an inadequate nursing staff. The number of patients to a nurse was given as thirty-eight, of whom some were epileptics and some sick. The patient on whom the recent inquest was held was an epileptic suffering from complete dementia. Œdema of the lungs was found to be the cause of death, and in giving a verdict to that effect the jury added their opinion that the staff of nurses in the lunatic department was insufficient, their hours of attendance too long, "and that insane patients should not be detained in the workhouse—a place never intended for their reception—but sent to the proper lunatic asylum, where there were proper means for their treatment." In summing up, the Coroner remarked that the inquiries held in the lunatic department had resulted in most of the recommendations of the juries being carried out. Although some verdicts had been objected to, yet the Local Government Board had succeeded in instigating the Guardians to attend to the advice given. The impropriety of pauper attendants for the lunatics was shown at one of the inquiries, where a man employed in this capacity was known to have been himself in jail two hundred times. The Coroner, Mr. S. Finnigan, has proved himself a powerful advocate for humane treatment of the insane, and his opinions on the inadequacy of the nursing in the lunatic wards deserve consideration.

### Minor Appointments.

North-Western Hospital, Haverstock Hill.—Miss Hedwig Ida Proschwitzky has been made Night Superintendent of this hospital. She was trained at St. Marylebone Infirmary, and was afterwards for three years head nurse at the Darlington Hospital. Miss Proschwitzky was sister at St. Saviour's Infirmary, East Dulwich, and then sister at the Greek Hospital, Alexandria, for eighteen months. We wish her success in her new work.

### A Great Movement: The Nurses' Co-operation.

### II.—SOME FINANCIAL ASPECTS.

A consideration not to be overlooked in the organisation of any company is the financial one. Before the Nurses' Co-operation could appear before the public eye, it needed a local habitation, with rent paid in advance. It required also certain officials whose salaries must be guaranteed. Some money in hand was needed for advertising, furnishing, postage, and printing, for all the miscellaneous details which surround the commencement of any movement. The financial difficulty was, fortunately, met largely from within. Miss Belcher and Miss Napper each advanced £100, Mr. W. H. Burns and Mr. Burdett guaranteeing them against loss of every kind; and on hearing of the aims of the association, Mr. W. Capel Slaughter made it a gift of £100. This was on March 30th, 1890, and on December 3rd of the same year a gift of a similar amount was announced from Mr. Charles Cheston. Thus with a capital of £400 the Co-operation started its career. An office was taken at 8, New Cavendish Street, Portland Place; this was opened in January, 1891, for the enrolment of nurses, and by February 1st the Co-operation was ready to supply the public. At that time it had thirty nurses on its register. Miss Honnor Morten acted as honorary secretary, and Dr. J. A. Goodhart as treasurer, while for superintendent the Co-operation secured Miss Hicks, who had till then been matron of the Sick Children's Hospital in Great Ormond Street, but who is perhaps best known to the public as Sister Philippa of the Egyptian campaign. The rules were largely founded on those of the Philadelphia Nurses' Directory. In the United States the nursing institution does not exist, and the directory tells the public where trained nurses may be found, and on what terms they work. It is necessary, however, to make clear our use of the words "register" and "directory." We do not pretend that the Nurses' Co-operation had a complete and exclusive register of the trained nurses of the United Kingdom. There was no suggestion that the nurses on its roll were better than those outside its walls, nor was there any suggestion that to be on the register was like possessing a medical degree, a thing which once obtained was a property for life.

Very rarely, indeed, but on one or two occasions, nurses belonging to the Co-operation have been asked to resign on account of complaints made by those whom they have been nursing. But the Co-operation guaranteed that the nurses on their register were trained and experienced. No one was entered on it who had not had at least a year's hospital training, followed by two years of private or district nursing. Each nurse stated the fee she was willing to work for, and she was not allowed to charge more, but the minimum fee registered was a guinea a-week. The Co-operation was not a charity; it was intended neither to employ the incompetent nor to supply the unfortunate. It was a business association to give good work for fair pay. Speaking generally, the fees charged were two guineas a week for ordinary non-infectious cases, and £2 12s. 6d. a week for special, infectious, mental, and maternity cases, a half-week being charged a guinea, and nurses for a day at the rate of 10s. 6d., whilst massage was charged 5s. 6d. a visit. The terms, in short, were those of most good institutions. The difference being that in the Co-operation the nurses received all they earned, minus a small percentage to pay office expenses. This percentage was fixed at 7½, that is, eighteenpence in the pound, a modest sum which most people would be glad to pay for the privilege of having their names brought before the employing public. The only restriction on liberty being that nurses were not permitted to refuse any case offered them through the Co-operation. But the amount of work a nurse obtained depended largely on herself. Persons sending for a nurse could choose one whom they knew and liked, or of whom they had heard a good report. In many cases it is important to the doctor to have a nurse who understands his methods, and who will neither omit important details nor waste his time by narrating trivial ones in her reports to him. Thus to become a member of the Co-operation did not mean the receiving of a fixed salary, large or small. Given the advantage of the central office, the registration, as a fit and proper person to take charge of the sick, the chance of coming in contact with doctors who would recommend her, the nurse's popularity and prosperity depended upon herself. But the advantages of belonging to the Co-operation were so great, that practically the committee could pick their nurses, and they were careful to justify the confidence of the public by choosing only the best.

Before the association began work 30 nurses were enrolled, but by the end of the first year the staff numbered 185. Between February 2nd and December 30th, 1,127 families were supplied with nurses. "In addition to this," says the executive committee in their first report, "about 30 cases were refused daily during the influenza epidemic last May, and at the present time we are refusing about 70 cases daily. We have sent nurses to America, India, Egypt, the Scilly Isles, and nearly every part of the Continent. We sent a nurse to Spain at the time of the Burgos Railway accident." Thus from the first the Co-operation had been a success. The public had welcomed this bureau which had at once put them in touch with good nurses. The Co-operation was, what every successful association must have proved itself to be, a distinct public convenience.

Had it been also a convenience to the nurses? The hundred and fifty who had not joined before it started work must have thought so, or they would not have been so anxious to join it. Since that date the staff has risen to 258 in number. Altogether 360 nurses have joined the Co-operation since it started, but resignations have taken place from various causes. There are, however, plenty ready to take the place of those who go, but the committee do not want to have on the roll a number of nurses for whom they cannot find work. That the Co-operation can provide its members with steady work is evident from the amount of the nurses' earnings. Take two examples. Nurse D., who joined the Co-operation on February 13th, 1891, had earned by the end of the year £86 12s. Nurse B., who joined on April 10th of the same year, gained by the end of the year £63 10s. 6d. Each had taken a sufficient holiday out of this time, and even allowing for the fact that they had to pay for lodgings and for board when not at work, it is evident that they were much better off than they would have been in any institution. While the Co-operation avoided anything akin to the methods of the so-called "Homes," it utilised a few rooms situated above the office for lodgings, or, rather, for a "nurses' hotel," which those could use who chose. This was much appreciated, and the receipts from this averaged £11 10s. a month. A sick-room for nurses suffering from non-infectious illness was maintained, and the nurses were generously attended by the honorary physician, Dr. Haddon, whose death, in May, 1893, was deeply deplored, and his successor, Dr. H. P. Hawkins, and the honorary surgeon, Mr. Bernard Pitts.

### Wants and Workers.

*Artificial Limbs.*—We submitted this question to a surgeon of eminence, who writes:—Very few instrument makers know anything about artificial limbs, and still fewer surgeons. No attempt is made to teach students about them. So far as I know the principal points are few in number and not complex, but I have always found that I must personally supervise the maker. Here then is an opening for a new league, "The League of Artificial Limb Wearers," banded together to arouse surgical and public opinion, and to secure reforms which must tend to immeasurably increase the happiness of very many people. Meanwhile our correspondent should consult a surgeon of eminence and get him to supervise the maker she may employ on his advice.

### Junius S. Morgan Benevolent Fund.

The objects of the Fund are: (*a*) To afford immediate pecuniary or other relief by loan or absolute gift to matrons, sisters, and nurses (if members of the Pension Fund) who may be in distress, and to assist them in keeping up the payments of premiums on any policies they may have taken out in the society; (*b*) to grant annuities to matrons, sisters, and nurses who, from no fault of their own, may be or are unable to provide for themselves after sixty years of age. Applications for grants, which should be addressed to the hon. secretary, are considered and dealt with at the quarterly meetings. No application can be considered unless the papers are completed at least fourteen days before any such meeting.

Report for the Year 1894.

The large number of nurses requiring the aid of the Junius S. Morgan Benevolent Fund in 1894, proves the wisdom of its generous founders in thus supplementing the scheme of the Royal National Pension Fund for Nurses. Sickness and difficulties of a temporary nature are liable to overtake the most provident of nurses, and the managers of the Pension Fund have ample proof that, in the absence of the assistance of the Benevolent Fund, many nurses would be compelled to withdraw from it—possibly never to rejoin it, or, in any case, only doing so at a greater cost to themselves.

It is the strenuous endeavour of all those connected with the administration to help nurses overcome by misfortune to remain members of the Fund which provides for them when working days are over.

To assist in this direction is one of the most useful objects of the Benevolent Fund. Nurses themselves feel this to be so, and seldom fail to express their gratitude when benefited. During the past year temporary aid was extended to sixteen nurses, chiefly in payment of premiums, the applicants being incapacitated by ill-health. In one case the nurse's illness was so long and so severe that a whole year's premiums were met for her, and pecuniary assistance was granted her besides. In most cases aid for at least three months is found necessary, in order to really benefit the nurse, and the committee are happy to report that instances seldom occur of application being made where the need is not very real.

Having regard to the large amount of temporary assistance required, the committee have to be careful not to overburden the Fund with life pensioners, who form a large and important class of those benefited. In the earlier days of the Fund's existence, these pensioners were received from the ranks of the older nurses, who, in their working days, had had no opportunity of joining any provident fund. The time has now come, however, when help is required to supplement the endeavours of those nurses who joined the Royal National Pension Fund as soon as the opportunity was afforded them, but too late in life to make adequate provision for their old age. Thus the ability of the committee to help those who have never been members of the Pension Fund must become more and more limited; indeed, it may be necessary at some future time to confine aid altogether to those within the Pension Fund. Already the Benevolent Fund has a roll of 25 life pensioners. Life pensioners are added to the list every year, and from the fact (which the committee are glad to record) that the ranks are seldom thinned, especial amounts have to be set aside to meet a steadily increasing expenditure in this direction.

Donations for special objects have again been granted during the past year, and three nurses have been helped in this way. Loans to members have also proved useful, and in 1894 five sums of money were devoted to this purpose. Many nurses prefer assistance rendered in this form, as detracting less from their independence when their need is only of a temporary nature. The committee's chief business is to administer the funds of the Benevolent Branch of the Pension Fund, but in the course of their work it is necessary to extend their help in other directions. Many requests for advice reach them from nurses, and they also procure much help, and even employment, for nurses through private influence. In short, the work of the Benevolent Fund is a far-reaching and increasing one. To show how large the work of the fund has become, it is only necessary to mention that forty nurses received assistance during the past year. In order that all really urgent appeals for aid may be met, a most judicious distribution of the income at command is necessary.

To enable the committee to aid the older nurses outside the fund, help will be most welcome. Those who owe a debt of gratitude to any nurse who has attended them in sickness, cannot better express their feeling than by placing a sum of money to the nurse's credit in the Pension Fund, and subscribing to the Benevolent Fund, that she may be adequately provided for in time of need.

The following cases, which show some instances of the manner in which help has been dispensed by the Benevolent Fund, may prove interesting:—

A Few Typical Cases.

Life Pensioners.—No. I. Nurse B. was left by the death of her husband unprovided for, and having been trained as a nurse she followed this calling for 19 years. She joined the Pension Fund at its commencement, and, being at the time over 55 years of age, she had, in consequence, to pay very heavy premiums. At the age of 63 years, before her full pension would be due, she found herself unable to continue her calling through failure of eyesight and general weakness. She bore the highest character amongst the patients whom she nursed; and in the neighbourhood where she carried on her work, friends volunteered to augment her savings, but, in spite of this addition to her income, her policy, converted into an immediate "annuity," was found insufficient for her wants. The Benevolent Fund, therefore, came to the assistance of this deserving case, and granted her sufficient to place her in comfort for the rest of her life.

No. II. The case of Nurse L. afforded the Benevolent Fund another opportunity of rewarding praiseworthy efforts to provide for old age. Nurse L. is now 69 years of age; she has nursed in a hospital for six years of her life, and thirty-six years have been spent in private nursing, during which time she was never in a position to lay by very much. Whilst able to work, she joined the Pension Fund. When applying to the Benevolent Fund for aid, it was found that she had been living on but a few shillings a week. The Fund, therefore, in consideration of her age, her character, and her wants, consented to provide for her for the rest of her life.

Help in Sickness.—Nurse W.'s case is a sad one. When only 27 years of age she was first attacked with rheumatic arthritis, in spite of which painful affliction she struggled on with her work until forced to relinquish it to enter a hospital. She then applied to the Benevolent Fund for aid as her resources were exhausted. (Like many other nurses, demands on her money were made by her family.) On leaving the hospital, somewhat better, in two months' time, the Benevolent Fund granted her 10s. a week for two months, that she might recruit her strength without anxiety, and it also paid her premiums to the Pension Fund, of which she is a member, for six months.

A Useful Loan.—Through illness and difficulties Nurse S. fell into arrears with the payment of her premiums to the Pension Fund. She was a hardworking nurse with but the limited salary of her calling to draw upon, yet she not only almost supported her aged and helpless father, but managed also to lay by in the Pension Fund for old age and sickness. Under these circumstances the committee of the Benevolent Fund gladly lent her the money she required to pay her premiums in arrears, to be paid back at her convenience.

Nurse Phœbe Kirk.

The committee regret to have to record the death of one of the beneficiaries of the Benevolent Fund, Nurse Phœbe Kirk, who died during the year after a painful illness. Nurse Kirk had not long joined the Pension Fund, having had up to a recent date many calls on her resources. As soon as these ceased she exercised great self-denial and put by a considerable sum of money. During her first severe illness the Benevolent Fund came to her assistance.

Mrs. Walter H. Burns.

The committee are glad to announce that Mrs. Walter H. Burns has kindly consented to join them in their work of distributing the fund, in which her family has taken so munificent a part by providing for the succour of nurses. Mrs. Burns is not only the wife of the chairman of the Royal National Pension Fund, but is the daughter of the late Mr. Junius S. Morgan, whose name can never cease to be regarded with gratitude by the generations of nurses who will benefit by his generosity.

In spite of the large number of calls on its resources during the past year, the financial position of the Benevolent Fund is satisfactory. During 1894 a sum of £425 19s. 5d. was expended in grants, &c., as against £375 13s. 4d. in 1893, and £238 1s. 2d. in 1892. The total sum standing to the credit of the Fund on December 31st, 1894, was £11,160 13s. 4d. (or exactly £100 more than at the end of 1893), of which amount £10,139 11s. is invested; the balance being loans to policy-holders, cash in hand, &c., as shown in the balance-sheet.

The committee hope that the fact of the fund not being in debt, as is the case with so many benevolent institutions, will induce many, able and willing to assist in so excellent a work, to send liberal subscriptions or donations.

(Signed) E. L. Rothschild, President.
Rosalind Pritchard, Hon. Sec.

28, Finsbury Pavement, E.C.

### Everybody's Opinion.

[Correspondence on all subjects is invited, but we cannot in any way be responsible for the opinions expressed by our correspondents. No communications can be entertained if the name and address of the correspondent is not given, or unless one side of the paper only be written on.]

### BURDETT'S OFFICIAL NURSING DIRECTORY.

"A Registered Nurse" writes: I see there is a query in last week's Hospital as to the relationship of C. Belasyse Myers to Mrs. Bedford Fenwick. Mrs. Myers is her sister, and Mr. Myers has the same initial as his wife. The lady, however, is known as the authoress of various little books.

### KIMBERLEY HOSPITAL, SOUTH AFRICA.

We have received the following letter, dated March 7th, 1895: "We have observed in *The Hospital* Nursing Supplement for February 2nd, 1895, certain remarks, in reply to an inquirer, reflecting on the Kimberley Hospital. The statement there made that matters are so unsatisfactory as to demand a Government investigation, pending which English gentlewomen had better not apply for the vacant post, is absolutely and maliciously false. The best reply we can make is to ask you to publish the names of the members of the Hospital Board of Management, which is constituted as follows:—E. A. Judge, Civil Commissioner, chairman; Justice Salomen, J. B. Currey, manager London and S. A. Exploration Company; C. M. Bult, J.P., registrar of natives; M. Cornwall, J.P., Deputy Sheriff, late Mayor of Kimberley; Government nominees, W. H. Craven, secretary De Beers Consolidated Mines (Limited); C. E. Nind, director De Beers Consolidated Mines (Limited); J. M. Jones, director De Beers Consolidated Mines (Limited); representing the mining boards. The Mayor of Kimberley, the Mayor of Beaconsfield; R. M. Roberts, secretary du Toits pan mining board, elected by the subscribers. These, together with the names of the medical men who sign this, are, we think, a sufficient guarantee to anyone who knows Kimberley that an English gentlewoman may fearlessly apply for any post in the Kimberley Hospital.

James A. Smith, F.R.C.S.E., L.R.C.P.E., Dist. Surgeon, Fellow Royal Physical Soc., Ed.

Arnold H. Watkins, M.D. Exam., M.R.C.S.

J. Edder Mackenzie, M.R C.M.

Henry Symonds, M.D., Lond.

Arthur Fuller, M.B., M.R.C S.

} Consulting Surgeons.

Alfred Ed. Thomson, M.D., M.R.C.S., Senior House Surgeon.

[We very gladly publish the above letter. The advice we gave to intending candidates for the post of matron to the Kimberley Hospital was in no way malicious, but founded on evidence in our possession which pointed to a state of mismanagement there which could alone be dealt with effectually by Parliamentary inquiry. It seems to us essential that this inquiry should take place before the vacant office of Matron is filled up. The Kimberley Hospital receives a grant from the Cape Government, and the board, or several of them, are appointed by the Government; hence the institution of such an inquiry as we demanded is undoubtedly the proper course for the Cape Government to take in regard to this institution. We readily believe that the present board and the medical staff at the Kimberley Hospital are most anxious that the management should be efficient. Unfortunately, gentlemen serving upon boards of this kind very often know little or nothing of the true state of affairs, so far as the internal work of a hospital is concerned. The confidential letters which have reached us describe a state of things which existed there at the end of 1894, and which rendered it quite impossible for us who represent the interests of all workers in hospitals to do less than warn those who contemplated applying for work at Kimberley not to do so until a public inquiry had taken place. Whilst, however, we desire to give the present board and staff of the Kimberley Hospital credit for their desire to secure efficiency, we must stand by our warning, and urge them to co-operate with the Cape Government in bringing about an immediate public inquiry at the institution and the necessary reforms.—Ed. *T.H.*]

### Novelties for Nurses.

### SWEETMEATS.

We can confidently recommend Messrs. Clarke, Nicholls, and Coombs' cream caramels to lovers of sweetmeats. They are most deliciously flavoured, and are free from that cruel adhesiveness which sometimes makes the consumption of caramels a pain rather than a joy.

### REMY'S PUDDING POWDER.

Remy's pudding powder gives an excellent opportunity to vary the diet of invalids. It is so very nice, and nutritious also, that it should certainly become a favorite ingredient in sick-room cookery. It can be used for puddings and cakes, and makes an excellent thickening for soups. It is guaranteed to be pure, and resembles closely the cornflour generally used. It possesses, however, qualities of its own, and is very delicate in flavour and easily cooked. The same firm supply a very good starch called the Royal Rice Starch. The London agent for both powder and starch is H. Becker, Lower Thames-street.

### AN EXCELLENT THERMOMETER.

Messrs. W. Bailey and Son, of 38, Oxford Street, are very quick to note useful novelties for nurses. Their latest speciality, the "half-minute" thermometer, will be hailed as a boon by the tired nurse who has to register the temperatures of a whole ward full of patients at the end of a hard day's work. Five minutes is a large amount of time to devote to each patient when as many as sixteen, perhaps, have to be observed. A short stay with each patient gives the thermometer a chance of a longer life, and, added to the price, which is very low, the "half minute" thermometer will prove a most economical purchase both for nurses and institutions.

### Appointments.

Birmingham and Midland Ear and Throat Hospital.—Miss Frances Marion Geard has been appointed Matron of this hospital. She recently held the post of assistant matron at the Warneford Hospital, Leamington.

Woolwich and Plumstead Cottage Hospital.—Miss A. E. Spooner has been appointed Matron of this hospital. She was trained at St. Mary's Hospital, Paddington, and was assistant matron at Cane Hill Asylum. We congratulate Miss Spooner on her appointment.

### Death in Our Ranks.

Mrs. Page, late matron of Hunstanton Convalescent Home, died on March 19th, respected and regretted by a large circle of friends. Mrs. Page retired at Christmas from the post which she had ably filled for twenty three years, in the course of which 10,000 patients had passed through her hands.

### American News.

### THE CONVENTION OF SUPERINTENDENTS.

The summary of the proceedings at the Superintendents' Convention at Boston was contained in our latest American letter, and we now give the address of welcome which was delivered by Miss Linda Richards. The full report of this highly successful convention was forwarded by the secretary of the society Miss Mary Littlefield, with a most courteous note, in which she says that it was the voice of the Convention of Training Schools Superintendents recently held in Boston, that a report of the proceedings should be published in The Hospital.

### Miss Linda Richards' Address.

It is now more than twenty years since, in this city, to which I now welcome you, I took charge of my first training school. Many are the changes which have taken place in the years which lie between that time and to-day. Perhaps in no one particular do we notice this more than in the revolution of feeling toward training schools and trained nurses. Then this work was all experimental, and as such, was looked upon with great distrust, and grave doubts were entertained regarding its success. Time has changed all that, and training schools have proved themselves among the greatest blessings of our land and are recognised as such to-day. Every training-school in New England feels honoured by having this association hold its second annual meeting in this city, and very hearty is the welcome which to-day I extend to you from each hospital and school in connection therewith. May we take time to look backward to compare your reception at this time with my own twenty years ago? The school of which I was to have charge had been in existence one year. In that time it had had two superintendents, each with some practical knowledge of nursing, but without having had training. These women had done the best they could, but what women with no training can be expected to properly manage a training school? Is it a wonder that the doctors had pronounced it a failure, and wished to return to the old order of things? Such was the case, and so discouraging was the report given by them to the board of trustees, that they were in grave doubts concerning the wisdom of giving it a second year's trial. But when it was put to vote it was, by the overwhelming majority of one vote, allowed one more year of grace, provided a trained nurse could be found to take it in charge. I was asked to come and help them, and did so. I came, not knowing into what I was coming. I was not met with outstretched hands of welcome; the doctors did not want me; the nurses who had framed their own rules and planned their own work, did not wish for a trained superintendent who, very likely, would change their ways into those of her own; the trustees, with the exception of the president of the board, left me to myself. Shall I ever forget or cease to be thankful to him for taking pains to call upon me to express his faith in the work and assure me of his willingness to render assistance in any way possible? The Training School Committee were very kind indeed, and gave me all needed support, but my days—yes, and many of my nights (for I often acted as special nurse to trying cases)—were spent among those who wished me in any place but the one to which my duty called me; not a very pleasant picture to look back upon, and I seldom recall it excepting for the purpose of contrasting it with the present. The plan of the work in the wards was so unique that I will give a little sketch of the duties of one nurse for five days. This will describe the duties of each, as it was a rotatory system. Nurse A. on Monday, had charge of the ward, attending to the duties of a head male nurse; on Tuesday she had entire charge of the food for the ward, with the usual rounds, also of pantry, washing all dishes, &c.; Wednesday she attended to the general cleanliness of the ward and linen closet; Thursday she stood at the sink in the bathroom till noon and washed poultice cloths and bandages—in the afternoon she slept, and went on night duty Thursday night. Her hours, when on night duty, were supposed to be from eight p.m. to seven a.m., but she reported for duty when she felt like doing so at any time before ten p.m.; on Friday she rested, to be ready to start the round again on Saturday. The nurses dearly loved this method, and bitter were the tears shed when the superintendent, whose training at the North-East Hospital, under Dr. Dimeck, had been thorough though limited, thought well to change it. After graduating from the North-East Hospital, and spending thirteen months as night superintendent, under the leadership of Sister Helen, whose wonderful executive ability placed the New York Training School, in connection with the Bellevue Hospital, upon its firm foundation, and as a result of this training, she thought the superintendent the person to plan, work, and make rules for the guidance of her nurses. This she proceeded to do: The changes were made; the trials accompanying such changes need not be mentioned, they are too well known to every superintendent to make a repetition necessary. The work moved on; soon more wards were given the school; then came a new class; women who were a joy and a pride—they have ever continued to be; to them was due very largely the entire change of feeling toward the training-school. Their faithful and intelligent work met with appreciation; their presence gave dignity to the place, and after a time smiles, instead of frowns, greeted us, and occasionally a word of commendation was heard, at first cautiously spoken, and before very long those who had at first looked upon us with disfavour, became our firmest friends, and when the school was declared a success, they spoke of it as "our school," and thought the idea of its organisation originated with themselves. They adopted it, and we rejoiced in having won friends from among those who once had seemed to be our enemies. These are only a few passing memories of one school. Others were soon organised in this city and in other cities in New England, each facing and conquering its own peculiar trials, and each in turn proving to be a wonderful blessing to the hospital with which it is connected. To you, who are superintendents of hospitals and training schools, is this wonderful change due. How vastly different are the hospitals of to-day from those same hospitals a score of years ago? A painful duty was a visit to most of them then. To-day a visit to those same hospitals is an inspiration. Visit Bellevue, Blackwell's Island, Tewksbury and many others. They all tell the same story. The perfect cleanliness and order of the wards, the homelike appearance, the contented faces of the patients, make even hospital workers wonder how so much can have been done; truly a wonderful work is this which we are permitted to take a part in. But the progress of the past twenty years is small in comparison to that which will be made in the twenty years to come. Training-school superintendents have a mighty work before them; there are perplexing questions to be settled, new branches of the work are to be opened, and new methods of doing the ordinary everyday work are to be thought out. Instruction in schools must be made more uniform, the standard must be raised, and upon the superintendents rests the duty of having these matters properly adjusted. Women at the head of training schools are to-day bearing great responsibilities; each one must feel this. The organisation of this association meant a great deal more perhaps than some of us realised. It means much for each member; the responsibilities will not grow less as time goes on; and in extending to you a welcome to-day I welcome you to the considering of very grave questions, the solution of deep problems which must take much thought, and will influence each school represented here, and through these schools all training schools in America. May the judgment of this society be sound, and its decisions wise; so shall we bring much good, not only to our own schools but to those not represented at this meeting.

## Figures and Tables

**Fig. 1 f1:**
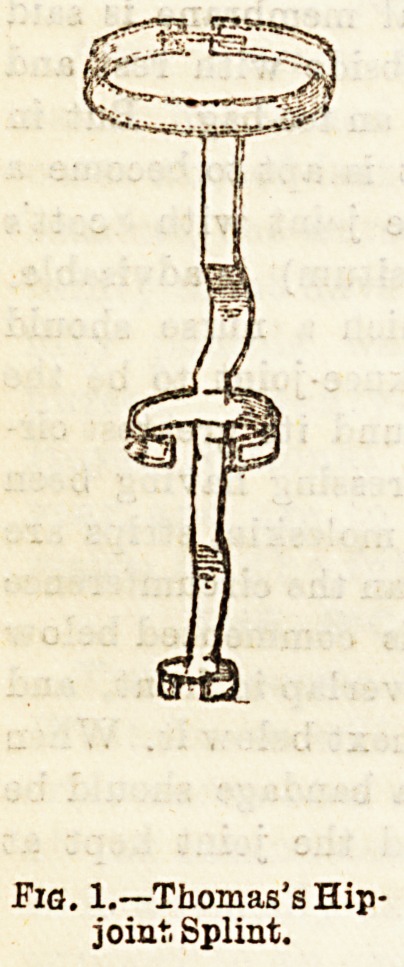


**Fig. 2 f2:**